# Sanitary Legislation

**Published:** 1858-01

**Authors:** 


### SANITARY LEGISLATION.

Reports on "Metropolitan Drainage," on the "River Thames," and "Metropolitan Improvements," lie before us. In the first report, which is the only one to which we need refer, the question of the utilisation of sewage is dismissed as too theoretical to be at present entertained by the Metropolitan Board of Works. The reporters, Messrs. Galton, Simpson, and Blackwell, are of opinion that the only practicable mode of disposing of the sewage is to provide for its rapid removal from inhabited districts, and for its collection in main outfalls, where private enterprise may be at liberty to utilise it; and that the best outfall for the north side of the metropolis is a place between Mucking Lighthouse and Thames Haven; and on the south side, Higham Creek, in the Lower Hope. They dwell on the increasing pollution of water streams throughout the country as an evil consequent on improved house-drainage, and suggest the necessity of attention to this all-important fact. With respect to the general question of the purification of the Thames, it is quite evident, say the reporters, that a large tidal navigable river passing through a country thickly inhabited must be polluted to a certain extent, from the refuse of ships, barges, and the like; but this contamination might, they think, be prevented in great part if the conservators of the river were invested with more power for protecting the river from being polluted. Reviewing all that is argued in this report, we opine that the plan for the grand scheme of London drainage proposed in the report, is the best yet offered of the kind; but we still think that it is not *the* plan, and that neither the plan nor the planner of this great national undertaking has as yet appeared.

